# Topics of Public Interest

**Published:** 1914-01

**Authors:** 


### TOPICS OF PUBLIC INTEREST.

---

DASHEEN, A NEW VEGETABLE. The name is said to be a corruption of de Chine (from China) from its similarity to the taros of China, Polynesia and Hawaii but it seems to be indigenous to tropic America. It is cultivated in Porto Rico and Florida, as well as various other parts of North and South America. Its cultivation is somewhat similar to the potato's, each hill containing one or two large corms (underground stems) aggregating five pounds or so and from these grow smaller tubers, weighing four to five ounces each and aggregating eight to ten pounds. The corms are used first as they do not keep so well as the tubers. Otherwise, both corm and tubers are practically the same and resemble the potato but are dryer and more nutritious, containing 3 per cent. of protein and 27 per cent. of carbohydrate as compared with 2.2 per cent. and 18 per cent. respectively for the potato. The dasheen may be cooked in the same variety of ways as the potato, including the use of pies and of breads from the dried and ground meal, for which the potato may be but is little used. The present price is five cents a pound, as compared with about two cents (at about $1.00 per bushel) for the potato. The normal price may be considered to be one cent a pound, the dasheen being worth about half as much again, from the standpoint of food value. There is no reason why it should not compete with the potato.

---

School Registration in Buffalo. Sixty-three thousand, six hundred and sixty-four are registered in public schools, 26,728 in private and parochial schools. In an average population, 19 per cent. are between the ages of 5 and 14 and, for each additional year up to early adult life, the percentage is about 1.9 per year. Thus, almost exactly 25 per cent. is included in the school ages from 5 to 18. The population of Buffalo is about 440,000, so that to provide for full grammar and high school education of every child, 110,000 should be registered. The actual registration is over 90,000, not to mention vacation and night schools. As many children finish the high school before they reach their eighteenth birthday, it is obvious that very nearly ideal conditions of education have been reached at least quantitatively.

---

The Animal Holder. Illustrations of this device are commonly used by anti-vivisectionists, as prima facie evidence, that anaesthetics are not employed. It occurs to us that it might be a good plan to have some of the antis try to dissect a dead dog without a holder of some form, when they will realize that the conformation of the back and the projecting legs present difficulties of a mechanic nature not encountered with human subjects. And while we do not advocate a cruel experiment, it occurs to us that even with the animal holder, any but the coarsest vivisection would be extremely difficult unless an anaesthetic were used. Moreover, without regard to considerations of mercy, in which the medical profession is not altogether lacking, but from a cold blooded, scientific standpoint, any severe painful impression reacts so violently on secretion and motor phenomena as to render a vivisection without an anaesthetic valueless. Of course, this argument does not apply to the physiologic study of sensation but it does apply to practically every other problem in physiology.

---

Against Fee Splitting. The following declaration was adopted by the American College of Surgeons:

"I hereby promise upon my honor as a gentleman that I will not, so long as I am a Fellow of the American College of Surgeons, practice division of fees in any form; neither will I collect fees for others referring patients to me; nor will I permit them to collect my fees for me; nor will I make joint fees with physicians or surgeons referring patients to me for operation or consultation; neither will I in any way, directly or indirectly, compensate anyone referring patients to me; nor will I utilize any man as an assistant as a subterfuge for this purpose."

We can see no ethical reason for some of these provisions, in fact there is much to be said in favor of the proposition that the various attendants on a case should share equally any risk of failure to collect, and that each should receive an amount proportionate to the time and skill expended, with no discrimination as to time of collection nor as to the scale of charges, with due reference to their value. There are many cases in which the whole professional work is a unit, and we believe that, as in the case of the late President McKinley, the unit charge should be made and the fee split, but honestly and openly. Otherwise, an undue preference is given to certain forms of professional work. And we trust that this statement will not be quoted in a garbled form to be interpreted as an advocacy of what is ordinarily termed fee splitting.

---

Contract Practice. We have received from the New Zealand Branch of the British Medical Association a blank form of contract of medical officers with lodges, etc., which obviates, to a large degree, the economic and hence ethical objection to contract work. It should be understood, however, that contract practice is so legalized in England and the dependencies that the problem is somewhat different to what it is in the States. So far as can be judged, this blank contains specifications which insure a reasonable payment for professional services and prevent abuse of privileges on the part of members and their families.

---

U. S. Civil Service Examination—Male Medical Assistant. The vacancy is in the Bureau of Chemistry, but eligibles may be appointed to other positions which may become available. The salary is $1,800. Applicants will not be subjected to formal examination but will be rated according to general education and medical training, practical or professional experience and fitness and publications or thesis. Form No. 304 should be secured and submitted by Jan. 12.

---

Osculatory Hospital Endowment. An Ohio hospital has raised $10,000. Twenty thousand men kissed six members of well-to-do and locally prominent families, standing in line for the privilege. The price was $1.00—the discrepant figures not being explained. Nice, sanitary and moral method, isn't it? If we were real crude we could suggest a more profitable scheme.

---

THE J. N. ADAM MEMORIAL HOSPITAL at Perrysburg is to be equipped for 15 additional patients, and a reservoir for fire protection will be built, to cost about $8,000. Dec. 1, 1913, there were 147 patients, the original provision being for 135. There were 20 patients on the waiting list.

---

RECENT ENDOWMENTS. Cornell Medical School, four millions from anonymous benefactor; Johns Hopkins, one and a half millions from the Rockefeller General Education Board, to establish the William H. Welch Endowment for Clinical Education and Research.

---

ELECTROCUTION, for which word we disclaim responsibility, has been legally adopted in Arkansas for crimes committed after June 13, 1913.

---

DRESS REFORM. Various women's organizations of Cleveland have declared in favor of modesty in dress. Barring extremes which are comparatively seldom seen among decent women, the present fashion does not impress us as being especially immodest. Why associate erotic ideas with either set of limbs? But low necked street dresses in winter display an unpleasant area of goose flesh and predispose to respiratory affections, while the hobble skirt not only makes the gait awkward and interferes with proper exercise but is, at times, positively dangerous.

---

DANCING. So many of our contemporaries are publishing editorials on the vulgarity of the modern dance that we venture to suggest that they are either a year or so behind the times or that they have made a peculiar selection of dancing places. We hold no brief for dancing in general, but would point out that at no time for many years has the variety of dances and figures been so great as they now are and as they were in the time of our grandparents, and that at no time since the general introduction of the round dance has the rhythm so closely approached that of the minuet, which the declaimers of the modern dance have usually upheld as ideal. Even the strenuous quadruple time dances, such as the barn dance, tango and one-step are not necessarily immodest, though somewhat sudorific and liable to be ungraceful, especially if the dancers are not expert. The worst dance that we ever witnessed was a quadrille, and any dance may be done decently or indecently.

---

### A Problem in Ethics.

*The Illinois Medical Journal,* the organ of the state society, publishes the following:

"Below is a fac-simile of a postal card received by Chicago doctors:

THE MAYO CLINICS.

> The Mayo Clinics at Rochester begin promptly at 8.00 a. m. The Great Western Limited leaving Chicago daily at 6.30 p. m. with sleeper for Rochester is the only train which enables you to be present at the commencement of the work.
>
> Best route for your patients, too.
>
> For tickets and berths call on or phone H. C. Hilbourne, General Agent Passenger Department, Chicago Great Western R. R., 62 West Adams street, Chicago. Phone Central 5269. Train leaves from Grand Central station, Fifth avenue and Harrison street.

"Will the Minnesota State Medical Association stand for this, or will the same brand of whitewash be applied as was used in the case of skillfully devised articles in certain lay magazines?"

Let us digress for a moment and present the same ethical problem in clinical form. About a quarter of a century ago there were two young doctors, well educated and of great promise. They held teaching positions in a medical college. We happened to meet one of them casually and were surprised at his introducing the subject of medical advertising. He felt, that older and more prominent men had an unfair advantage in securing work and was seriously considering press advertising. We pointed out the disadvantages of this method and also the fact that, with increasing years, the unfairness mentioned would gradually disappear. However, he induced his colleague to enter the field of commercial medicine with him and ,naturally enough, they were expelled from the college with considerable notoriety. One newspaper sarcastically headed the notice: "This advertisement won't cost them anything." One of the two men is a case that has been lost sight of, to follow clinical dialect. The other eventually discovered that unethical advertising did not pay and he gradually returned to ordinary, ethical practice, but his prestige was gone and, not to enter into details, which were not particularly interesting, he died some time ago after a rather sad and hard life. What impresses us most forcibly in this case and in the majority of other similar ones that have come to our attention is that the excuse usually offered by a young man engaged in quackery is the unfair advantage taken by more prominent men in utilizing their social and professional standing in an adroit way so as to secure all the benefits of publicity without violating the letter of the code of ethics.

This much ought to be said on the other side of the question: "Unto him that hath shall be given," or, more vulgarly, "It is the fat pig that gets grease rubbed into its hide," is a general economic law as wide as the world and as old as history. The man who has achieved prominence gets a good deal of gratuitous advertising, often in objectionable ways and often without any personal responsibility. We are inclined to believe that the Mayos are in no way responsible for the postal card to which our contemporary takes exception, but that it is due to the enterprise of the passenger agent. Last year we refused to endorse the action of a Baltimore society which proposed that all surgeons whose names were connected in the public press with reports of operations or even of the removal of a patient to a hospital, should be brought to trial before boards of censors. This refusal was due not to any lack of sympathy with the general principle of ethics involved, but to the fact that experience has shown that, however modest a surgeon may be, there are customs of reporters and a demand for details on the part of the public that make it practically impossible for any medical man to avoid publicity of this sort. If we had endorsed editorially the resolution mentioned, we should have been compelled to condemn some prominent friend in almost every subsequent issue of the JOURNAL, or else to have been inconsistent.

But, it is worth while to consider seriously how far wealth, influence and business sagacity of unusual order shall modify the ethical conceptions of the profession so that, in the practical workings of the code of ethics, there is one law for the big man and another law for the little man. We make no claim to being able to solve the problem offhand. One reason is that it is difficult to draw lines. There is, for example, no objection to reporting or demonstrating a case before one's colleagues; none to the holding of clinics open to the profession by those fortunate enough to have hospital services of sufficient magnitude; but when some great man travels with a troop of assistants and trained patients and his demonstrations are placarded on the front of a theatre in ink that the laity as well as the profession assembled in that particular city can read, a great many of the humbler members of the profession break the tenth commandment and make unkind remarks. So, too, in the field of medical literature, there is no objection to the publication of one's observations, research and reflections; none to sending reprints; it is practically necessary for financial reasons, and desirable otherwise, that practicing physicians should have the editorial control of medical journals; no serious objection has thus far appeared against the publication of one man's clinical work in either book form or as a periodical; but there is no question but that the regular issue of such a periodical is a valuable means of publicity and it is highly probable that the same general policy of issuing bulletins will extend to others less worthy, and mainly for commercial ends. There can be no objection to the institution of hospitals and to the selection of competent practitioners to give their services to the patients; but the fact that such service has not been within the reach of the majority of the profession, that it involves a return of great value, and that selection has not invariably been made impartially, has led to the establishment of an unnecessary number of rival institutions in which the commercial interest of the physicians has been pretty frankly admitted. And, right here, let it be said that there is nothing essentially unethical in frank commercialism, either in a hospital, dispensary, medical school or any other professional enterprise. The ethical infraction consists in exploiting personal ends under cover of charity, philanthropy, and science, in securing special privileges and in making selections to desirable positions which obviously are limited to a very few, on other bases than merit.

We live in an age of commercialism. Professionalism has discarded some of its former ideas depending on an assumption of superior dignity, but the general principles of professional ethics must be maintained. They can not be maintained if they do not apply impartially to all. As stated, the highly efficient publicity which some men manage to secure by an adroit insistence on the letter of the code of ethics and by making use of personal advantages, is one of the greatest reasons for quackery. The profession must solve this problem with justice to all—not forgetting the justice of accrued earning capacity of any kind of capital, including prestige honestly gained.

---

**Centenarians.**

Andrew Jaszeakiewicz, a Pole, died in Buffalo April 17, aged 109.

Dennis Snow of Weehawken died on his hundredth birthday, May 27. He had worked 50 years as a railroad flagman and had accumulated a fortune of $50,000.

Robert Crichton, born in Perthshire, England, April 3, 1812, is still alive and in good health except for failing vision, in Caterham, 20 miles from London. He does not smoke and is nearly a total abstainer from alcohol, but uses snuff occasionally. He is a bachelor.

Frederick Halsey Janson, who died recently at Hove, England, is said to have been born on Feb. 8, 1813. He was the oldest solicitor in the United Kingdom, having been admitted to the bar in 1835.

Mrs. Nancy C. Rogers, who died recently at Montville, near New London, Conn., is said to have been born in August, 1809, in Groton, Conn. She was never ill until six months before her death.

Mrs. Louise Waterman Carpenter, of Worcester, Mass., is said to have been born on Aug. 26, 1806, in Michigan.

Mrs. Eliphalet Dorr, of Milton Mills, Me., is said to have been born on May 6, 1813, in Acton, Me., and celebrated recently her supposed centennial anniversary.

Benjamin Priest, of Canaan, Me., is said to have been born on May 12, 1812, and is reported to be still in excellent health and able to do light work about the farm.

Thomas Spillane, of Weymouth, Mass., who is locally reputed to have been born in 1812 in Ireland, has recently had pneumonia but is now convalescent.

Mrs. Jane Rich., a respected resident of Mount Vernon, N. Y., died from cardiac disease on May 18, at the age of 100 years and one month.

Bartholomew Gulguski, a native of Austria, died on May 19 in Brooklyn, N. Y., at the alleged age of 104 years.—*Boston M. & S. Journal.*

---

WOOD ALCOHOL. The other side. While recent articles appearing in the daily press have greatly exaggerated the danger in using wood alcohol, there is no question that the misuse of this material is a menace not only to life, but more terrible still, to eyesight, and this cannot be too strongly impressed upon the medical profession and laity. It should be understood, however, that with proper precaution it is not only a most valuable chemical, but is no more dangerous than many other solvents in ordinary commercial use.

Prof. Chas. Baskerville, chemist of the N. Y. State Factory Investigating Commission, and probably the greatest living expert on the properties and uses of wood alcohol, praises its value to the chemical and industrial world and states that it is dangerous only when taken internally or when its concentrated vapors are breathed for long periods in inadequately ventilated spaces.

In our December issue we referred to a case of blindness caused by the use of wood alcohol shellac in a Brooklyn brewery, the workmen applying the varnish to the inside of beer vats where adequate ventilation was impossible without some device for purifying air. The information in this instance we obtained from a circular from the N. Y. Committee for the Prevention of Blindness, released for publication Oct. 27, 1913, and was headed "Another Victim of Wood Alcohol." We have since been informed that the accident occurred in 1911 and that brewers are now so well informed as to the danger of using wood alcohol varnish on the interior of beer vats that its use for this purpose has been practically discontinued.

*The Paint and Varnish Record,* published in Chicago, under date of Nov. 15, 1913, claims that in the last forty years less than a dozen cases can be found in which death or blindness from wood alcohol has occurred unless the alcohol has been taken internally, and a recent booklet published by one of the large refiners of wood alcohol contains letters from twelve of the largest consumers of their products in the United States, these letters stating that none of their employees have ever been injured by its use.

There has been a great deal of discussion in regard to the external use of wood alcohol, and while personally we have encountered many individuals who have used wood alcohol as a liniment we have always warned them as to the possible danger and have urged the substitution of grain alcohol in spite of its difference in cost, and we believe it is the opinion of experts that the use of wood alcohol in any medicinal preparation for either external or internal use should be prohibited. We have used wood alcohol for many years as an excipient for shellac, to cleanse the skin before drawing blood or making a hypodermatic injection, for cleansing microscope cover glasses and to some extent as a solvent for color indicators; recently, also as an antifreeze mixture. With due precautions as to ventilation, we have had no ill effects from such use, personally or as observed in others as, for instance, in men varnishing floors.

It should be remembered that wood alcohol is one of a number of poisonous substances commonly used in the arts and trades, with perfect safety, so long as proper precautions are observed. The use of this substance, even as methylated spirits, in any drug, flavoring extract or beverage, should be absolutely prohibited, whether known by its chemical name or otherwise, and to prevent mistakes there should be a uniform law requiring the use of the well understood name, "Wood Alcohol" and a warning against its internal use, on any container whatever, placed on the market either wholesale or retail.

It is plain also that legislation should provide for the warning of workmen against the dangers of breathing the concentrated fumes of wood alcohol and for proper ventilation of all rooms in which it is employed.

As in the case of measures for safety against internal poisoning, legislation should include the proper branding of wood alcohol and, this necessarily opposes the interests of certain firms which profit in one way or another from the use of fancy names. Generally speaking, however, the wood alcohol industries recognize the ultimate benefit of marketing wood alcohol under its true name and there is developing a healthy sentiment that any object of commerce must sell on its own merits, under its own name, and without the benefit of mystery or disguise.

It should be remembered that the possible substitutes for wood alcohol are by no means devoid of dangers of their own. Grain alcohol has caused many more deaths than has wood alcohol, although its poisonous effects are so much more gradual. Gasolene and allied products from petroleum are by no means lacking in toxic properties and are of even greater danger on account of their inflammability and explosibility. Turpentine is highly toxic. One of our patients could not work three days in a shop where turpentine was employed without developing haematuria. With similar opportunities for exposure to wood alcohol, no death, blindness or similar serious lesion has occurred, so far as we have learned.

We have just received the thirty-seventh annual report of the Buffalo Eye and Ear Infirmary, and note that this institution has treated during the past year 1,652 cases of blindness and diseases of the eye, not one of which is ascribed to wood alcohol. We quote from their report: "By far the larger part of the disease of the eye from which these poor people suffer is preventable and therefore unnecessary. * * * It is high time that the public should begin to learn as medical men have long ago that venereal diseases far out-rank all the rest as destroyers of sight and hearing. * * * A second and distinct class are those due in some way to the use of alcohol. * * * It is probable that alcoholism with venereal diseases and tuberculosis, will long continue to be the great scourges of the human race."

However strongly we may advocate the control of the use of wood alcohol, and education against its dangers, it must not be forgotten that practically all fatalities and cases of blindness have been due to flagrant misuse and that the majority of these have been due not to carelessness in its commercial use but to its use as a beverage by the most reckless and degraded tipplers. There is, as far as we know, no case on record which could not have been prevented by reasonable precautions which would not interfere with any ordinary economic use of this product-barring, of course, those analogous to accidental death by various other poisons, and preventable only by the entire disuse of all such substances. And, furthermore, there is scarcely any industrial danger which presents so small a list of death and misfortune, as that connected with wood alcohol.

---

REPUTABLE MANUFACTURING PHARMACISTS DO NOT FURNISH EMMENAGOGUES FOR IMMORAL PURPOSES. Recently one of the leading manufacturing pharmaceutical houses received a letter upon the letterhead of a retail druggist, but signed by another name followed by the word "druggist." The person signing the letter may have been a clerk or successor of the druggist. The letter was as follows:

"There is practically no sale for your Emmenagogue Improved Pills, as few ladies know anything about them, and we can give no advice, as we know nothing about them ourselves as to dose, etc. Please let us know by return mail and tell us how to use, dose, etc."

Reply was made to the pharmacist whose name was on the letterhead, and was as follows:

"We have our doubts about Mr. ———— being a druggist, for we cannot imagine any druggist not knowing that it is not only immoral, but criminal, to sell an emmenagogue except upon a physician's prescription. We believe that every druggist who sells an emmenagogue direct to the consumer is put upon his notice that it will be used for an immoral and criminal purpose. Emmenagogues on our list are intended exclusively for the prescription trade and we never knowingly sell them for popular use or to be recommended and resold as remedies for female complaints, etc."

---

NUMEROUS CHANGES have been made in the staff of the Auburn City Hospital. As now constituted the staff consists of:

Consulting surgeon, Dr. F. H. Parker; consulting physicians, Dr. J. D. Tripp, Dr. J. M. Jenkins; consulting neurologist, Dr. F. Sefton; surgeons, Dr. J. P. Creveling, Dr. L. Heazlit, Dr. D. F. Armstrong, Dr. L. F. O'Neill, Dr. W. H. Coe, Dr. T. F. Laurie; physicians, Dr. E. G. Woodruff, Dr. S. W. Day, Dr. R. C. Almy, Dr. M. P. Conway; alternate, Dr. O. B. Swayze; homeopathic staff, Dr. E. W. Hitchcock, Dr. G. B. Mack, Dr. A. E. Baker, Dr. F. M. Hyatt; obstetrician, Dr. G. C. Sincerbeaux; radiologist, Dr. S. E. Austin; ophthalmologist-otologist, Dr. G. W. Whitney, Dr. F. A. Lewis; pediatrician, Dr. Amelia W. Gilmore; pathologist, Dr. H. I. Davenport.

---

Death from Wood Alcohol. Mistaking a jug of wood alcohol for whisky, a man in Buffalo took a considerable quantity on Nov. 27 and died.

---

Opposition to Eugenic Law. Physicians of Wisconsin object to the recently enacted law on the ground that the fee for certificate, $3 represents an inadequate examination. One physician points out that the Wassermann test, repeated four times, followed by a Noguchi test, and if that proved negative by the examination of spinal fluid should be the routine which would, of course, entail a considerably higher cost. It occurs to us that it is scarcely feasible to place pre-marital examinations on this plane of thoroughness. All that can be reasonably expected is a fairly careful clinical examination, to weed out conspicuous cases of physical and mental defect. An elaborate exclusion of syphilis, tuberculosis, etc., in latent stages would really not be worth while considering that it is perfectly possible for these and other diseases to be contracted after the issuance of the certificate. Neither do we conceive that the scope of the eugenic movement contemplates pelvimentry, and a thorough study of potential pathologic conditions in the pelvic organs of virgins.

---

Automobile Casualties. According to Dr. Franklin C. Gram, Registrar of Vital Statistics for Buffalo, out of 144 accidental deaths due to traffic, for the city, in the ten months up to Nov. 1, 1913, 69 were due to railroads, 29 to trolleys, 24 to automobiles, 19 to horse and wagon, 3 to motor cycles. A man has recently been killed by a slowly moving automobile near the city line, the driver being blinded by the headlight of a suburban trolley. The first conviction of an automobilist for carrying too brilliant headlights in New York City has recently been made, the judge holding court in the street in order personally to verify the statement of the policeman who made the arrest. Last month we witnessed a collision of an automobile with a post on account of the dazzling headlight of a standing auto. Having personally been subjected to considerable nerve strain on this account, we took the liberty to infringe on the privilege of the man who sustained the damage and to make a few appropriate remarks.

Many persons fail to understand that the majority of automobilists are the warmest supporters of proper legal control of the reckless ones. We have quite failed to convince one gentleman that drivers regard the policeman at a crowded corner as a friendly and authoritative guide and not merely with the fear of a would-be criminal confronted with an officer of the law.

There is a general misapprehension, too, of the factors of danger in traffic, the average person thinking of speed as the principal source of danger. As a matter of fact, a vehicle moving at a very slow speed is, on the whole, more dangerous than one moving at 25 miles an hour, with adequate brakes. The unlighted vehicle—and drivers of wagons are now protesting against carrying lights, which are a form of life insurance for themselves—the vehicle on the wrong side of the street, the slow vehicle in the middle of the street, the one that makes unexpected turns and stops are greater sources of danger than the one going at reckless speed.

Another cause of accidents is the impression of the pedestrian that he has the right of way. We do not question the propriety of the common law in this regard, but there are limits to what the most skillful and conscientious driver can do in the way of dodging children at play and adult somnambulists. As a rule, it is better to be alive after having conceded a privilege than to be dead after having insisted on one's rights, especially when the court will decide that, on account of contributary negligence and unavoidable conditions, the killing was neither a criminal offense nor a just ground for the collection of damages by one's heirs.

---

HOSPITAL FOR WESTFIELD. A vote of taxpayers carried, 209 to 173, the proposition to accept the gift of $10,000 from Dr. Charles E. Welch and of a site from Mrs. Josephine Dodman, these donations insuring the erection of the hospital, the village to assume the responsibility for its further maintenance.

---

DENTAL DISPENSARY FOR BUFFALO. Health Commissioner Fronczak will recommend the establishment of a free municipal dispensary when the new Common Council assembles. Rochester has three such dispensaries.

---

THE PANAMA-PACIFIC INTERNATIONAL EXHIBITION, to be held in San Francisco in 1915, will have a fully equipped emergency hospital, under charge of Dr. R. N. Woodward of the U. S. Public Health Service.

IMPROVED METHOD OF RECTAL ANAESTHESIA. J. T. Gwathmey, before the N. Y. Society of Anaesthetists, Nov. 20, 1913, described his method of anaesthesia by rectal injection of 55 per cent. of ether in olive oil, 1 ounce to each 20 pounds of body weight or, say 8 ounces for adults. Morphine is given hypodermatically one hour previously. Grile's requirements of innocuous association are met, and the patient may not even know that he is being anaesthetized. Respiratory failure, indicating too much ether, is met by washing the rectum with cold water. While the author makes no sweeping claims, there is good reason to hope that this method marks a new epoch in surgery.

THE U. S. TROOPS on the Mexican frontier are free from typhoid, on account of perfect camp sanitation and vaccination.

IODINE was isolated by Bernard Courois in 1911, his report to the French Academy of Sciences being made 100 years ago.

TELEPHONE AND OTHER PUBLIC UTILITY CONTRACTS. Warning: Do not surrender any contract in force for a new one unless you are positive that the new one gives at least as favorable a rate and that it is subject to renewal.

THE COPYRIGHTED RED CROSS. The Red Cross Society has protested to the A .M. A. and other organizations against the use of this insignium by anyone else, for instance in the form of marking a physician's automobile. It is said that the federal law provides a fine of from $1 to $500 and up to a year's imprisonment. Without being especially anxious to label ourselves as physicians, it may be said that the Maltese cross, of varying degrees of thickness, is a fairly ancient symbol—indeed long antedating Christianity and found on prehistoric pottery from various parts of the world—and that the choice of color must have been various and have included red, long before the Red Cross Society was heard of.

